# The impact of the COVID-19 pandemic on health-related quality of life of cancer patients in British Columbia

**DOI:** 10.1186/s41687-024-00759-z

**Published:** 2024-08-23

**Authors:** Sara Izadi-Najafabadi, Helen McTaggart-Cowan, Ross Halperin, Leah Lambert, Craig Mitton, Stuart Peacock

**Affiliations:** 1 BC Cancer Research Centre, Vancouver, BC Canada; 2 Faculty of Health Sciences, Simon Fraser University, Burnaby, BC Canada; 3 BC Cancer, Kelowna, BC Canada; 4 Department of Nursing and Allied Health Research and Knowledge Translation, BC Cancer, Vancouver, BC Canada; 5 School of Nursing, University of British Columbia, Vancouver, BC Canada; 6 School of Population and Public Health, University of British Columbia, Vancouver, BC Canada

**Keywords:** Cancer, COVID-19 pandemic, Quality of life, Survey, Telehealth, Health related quality of life

## Abstract

**Background:**

The COVID-19 pandemic resulted in unprecedented changes to cancer care in many countries, impacting cancer patients’ lives in numerous ways. This study examines the impact of changes in cancer care on patient’s health-related quality of life (HRQL), which is a key outcome in cancer care. The study aims to estimate patients’ self-reported HRQL before and during the pandemic and identify predictive factors for their physical and mental wellbeing.

**Method:**

The study employed the large-scale Outpatient Cancer Care (OCC) Patient Experience Survey, including the Veterans RAND 12-Item Health Survey, to evaluate cancer patients’ experiences and HRQL before (January to May 2020) and during the COVID-19 pandemic (May to July 2021). Paired t-tests were conducted to compare differences in Physical Component Scores (PCS) and Mental Component Scores (MCS) before and during the pandemic. Multivariable linear regressions were employed to investigate the factors (sociodemographic, clinical, and patient-reported experience) influencing PCS and MCS during the pandemic.

**Results:**

PCS decreased significantly during the pandemic, while MCS remained stable. Lower PCS contributors included older age, more telehealth visits, self-reported hospitalization, and a longer time since the last cancer diagnosis. Higher PCS was associated with urban residence, higher MCS during the pandemic, and perceived active Healthcare Provider (HCP) involvement. For MCS, lower scores related to female gender and more telehealth visits, while higher scores were associated with being white, higher education, high MCS before the pandemic, and perceived active HCP involvement.

**Conclusion:**

The OCC Patient Experience Survey provides a unique patient level data set measuring HRQL pre- and post- the onset of the COVID-19 pandemic. The study highlights challenges faced by cancer patients during the pandemic, with a significant reduction in PCS. However, the stability in MCS suggests effective coping mechanisms. Sociodemographic, clinical, and telehealth-related variables play a complex role in shaping both PCS and MCS. Perceived HCP involvement emerges as a crucial factor correlating with higher PCS and MCS. Navigating the post-pandemic era necessitates interventions fortifying patient-provider relationships, optimizing healthcare support systems, such as telehealth services, and prioritizing mental-well-being given its impact on both PCS and MCS.

## Abstract (plain language summary)

This study delves into the impact of changes in cancer care and COVID-19 measures on the well-being of cancer patients. As cancer care shifted during the pandemic, we aimed to understand how it affected patients’ quality of life. The study discovered that physical well-being significantly decreased, especially for older patients with more telehealth visits, while mental well-being remained stable, indicating effective coping strategies. Factors like the number of telehealth visits, healthcare provider engagement, and pre-pandemic mental health status played pivotal roles in shaping the mental and physical well-being of cancer patients during the COVID-19 pandemic. This study emphasizes the challenges faced by cancer patients during the pandemic and highlights the importance of supportive interventions for patient-provider relationships and prioritizing mental-wellbeing in the post-pandemic era.

## Introduction

The clinical management of COVID-19 placed significant pressure on capacity in health systems across the world. In British Columbia (BC), this had important implications for cancer care in the province [1]. COVID-19 profoundly impacted clinical workflow and timely access to patient care within BC Cancer Centres and Community Oncology Networks (CON) sites [2]. Cancer patients experienced delays in diagnosis, postponed tests, and treatment disruptions, leading to heightened uncertainty about future care [3, 4].

While the COVID-19 pandemic presented challenges for cancer care, it also propelled the use of telehealth to enhance the delivery of cancer-related services. The pandemic-induced shift in cancer care delivery has underscored the significance of accommodating individual patient needs and preferences. Recent findings have shown that that individuals with stronger mental health and those residing in rural areas are more inclined to value and continue utilizing these services even beyond the pandemic, potentially paving the way for even more tailored and patient-centric treatment plans in the future [5].

Additionally, modified cancer care, along with public health measures to slow the spread of COVID-19, undoubtedly impacted patients’ health-related quality of life (HRQL) over the course of the different waves of the pandemic [6]. Understanding the HRQL of cancer patients is crucial as it represents a key outcome in cancer care [7, 8]. The objectives of this study are two-fold. First, we estimate cancer patients’ self-reported HRQL before and during the COVID-19 pandemic. Second, we identify the factors that predict cancer patients’ physical and mental health, including how patients’ HRQL changes in the face of specific changes in care delivery such as the move from in-person to telehealth-based care.

## Methods

### Survey design

The Outpatient Cancer Care (OCC) Patient Experience Survey [7] was developed to inquire about cancer patients’ experiences with treatment, healthcare providers, and telehealth in the past six months. Additional items asked patients about their cancer diagnosis, treatment, and HRQL, measured using the Veterans RAND 12 Item Health Survey (VR-12) [8].

The OCC Survey was administered in multiple waves to patients undergoing active cancer treatment at a BC Cancer Centre and/or at a CON site. Survey Wave 1 collected data from patients who received treatment between April to June 2019; it was administered October to December 2019. Survey Wave 2 collected data from patients receiving cancer treatment between July to October 2019; this survey was administered January to May 2020. Patients who participated in Wave 2 were invited to complete a modified OCC Patient Experience Survey between May to July, 2021; this survey is referred to as Wave 3. In brief, the focus of Wave 3 was on patients’ experiences with telehealth and experiences with healthcare providers during the COVID-19 pandemic [5]. Specific questions inquired about patients’ perceived level of involvement of their healthcare provider and their assessment of cancer services received during the pandemic; a copy of the survey is available upon request. This current study focuses on patients’ responses from Survey Waves 2 and 3.

### Study participants

To qualify for the study, participants were required to be alive as of April 30, 2021, and to self-report receiving cancer care for any type during the COVID-19 pandemic. For this study, the onset of the pandemic is defined as March 16, 2020, marking the transition of in-person cancer care to telehealth by BC Cancer in response to the pandemic. BC Cancer, a leader in cancer care, collaborates with all health authorities in BC—regional organizations responsible for delivering health services—to ensure comprehensive cancer care, covering prevention, research, and treatment. Patients were given the option to complete the Wave 3 survey either online or in paper format. Patients were provided with the opportunity to complete the Wave 3 survey either online or in paper format. Each participant received an invitation letter via mail, which included a link to the online survey. Additionally, a paper version of the survey, along with a postage-paid envelope, was dispatched to those who had not completed the internet survey within two weeks. The survey was available in English and, upon request, in three other languages including Punjabi, traditional Chinese, and simplified Chinese.

### Data sources

The patients’ schedule and appointment records from the population-based BC Cancer Registry were accessed to understand their healthcare utilization (all cancer diagnoses in BC are recorded in the Registry). Registry records also include detailed data on every interaction the patient had with their healthcare provider at a BC Cancer site and/or a CON site.

## Analysis

### Study sample description

Using Survey Wave 3 responses, the study sample was characterized in terms of current age, sex defined at birth, education, race, health authority, region of residence, and tumour type. For any missing information, values reported in Survey Wave 2 were used. Categorical variables were summarized as the proportion of the sample within each group and continuous variables that were normally distributed were summarized as means and standard deviations (SDs). The physical component score (PCS) and mental component score (MCS) in the VR-12 are composite scores calculated based on a weighted combination of individual responses to various questions within the survey. These composite scores provide a summarized representation of an individual’s overall physical and mental health status, where higher scores represent better physical and mental health, respectively [8].

### Missing data

The level of missing data was assessed for all variables of interest. Little’s missing completely at random (MCAR) test determined whether the data was missing completely at random or not [9]. If the p-value from the Little’s MCAR test is greater than a chosen significance level (often 0.05), it suggests that the data is MCAR. A multiple imputation was conducted for each variable with missing values. As a rule of thumb, the number of imputations was set at twice the maximum percentage of missing data cases for the included variables to enhance the robustness of the results.

### Physical and mental component scores

A paired t-test compared the patients’ PCS and MCS between Survey Waves 2 and 3. Multivariable linear regressions were conducted to determine the factors that influenced patients’ PCS and MCS during the pandemic. For both regression models, sociodemographic (e.g., current age, sex, race, education level, health authority where care was received, region of residence), clinical (e.g., presence of more than one cancer diagnosis, number of telehealth appointments, time since diagnosis), and experiences (e.g., perceived involvement level of their healthcare provider during the pandemic and perceived level of cancer services received during the pandemic) variables were tested as independent variables. For the PCS model, two additional independent variables were included: hospitalization in the past six months (yes/no) and received chemotherapy during the pandemic (yes/no).

Statistical tests of associations were completed using SAS, version 9.4 (SAS Institute Inc., Cary, NC, USA). SPSS was used to assess data missingness pattern using Little’s MCAR test. Using R, multiple imputation (MICE package, version 3.14.0) and multivariable linear regression analyses (version 4.1.2) were conducted. Statistical significance was defined at *p* *≤* 0.05.

## Results

### Study participants

A total of 4733 patients were invited to complete Survey Wave 3; of which, 2612 patients responded to Wave 3 (response rate of 55%). Among patients who completed Wave 3, 56% opted for the paper survey, while 0.44% chose the online survey. Of those, the following patients were excluded: deceased as of 30 April 2021 (*n* = 12); missing diagnosis data from the BC Cancer Registry (*n* = 69); did not receive cancer care based on the scheduling data (*n* = 835); self-reported to not receiving cancer care (*n* = 293); and received cancer care outside of BC (*n* = 1). Six patients completed the survey twice; duplicate responses were removed randomly. The final analysis set included 1396 cancer patients (Table 1). The mean (± SD) age of the patients was 70.0 (± 10.8) years. The majority of the sample were female assigned at birth (*n* = 761, 55%) and white (*n* = 1119, 81%).

**Table 1:** Characteristics of study patients

Characteristics	*N* (%)
**Age**	
Mean (SD)	69.98 (10.81)
Min-Max	29–98
**Sex assigned at birth**	
Female	761 (55%)
Male	635 (45%)
**Education**	
8th grade or less	44 (3%)
Some high school but did not graduate	102 (7%)
High school or high school equivalency certificate	316 (23%)
College, CEGEP or other non-university certificate or diploma	393 (28%)
Undergraduate degree or some university	286 (21%)
Post-graduate degree or professional designation	244 (18%)
Missing	11 (1%)
**Race**	
White	1119 (81%)
East-Southeast Asian	164 (12%)
Mixed	32 (2%)
South Asian	17 (1%)
Indigenous (First Nations, Métis, Inuk/Inuit)	14 (1%)
Latino	11 (1%)
Middle Eastern	10 (1%)
Black	6 (0.4%)
Missing	23 (1%)
**Health authority**	
Fraser health	398 (29%)
Vancouver Island health	392 (28%)
Vancouver Coastal health	297 (21%)
Interior health	254 (18%)
Northern health	55 (4%)
Region of residence*	
Urban	1289 (92%)
Rural	107 (6%)
**Tumor type**	
Breast	431 (31%)
Prostate	272 (19%)
Lung	69 (5%)
Multiple myeloma	69 (5%)
Leukemia	53 (4%)
Non-Hodgkin lymphoma	50 (4%)
Cervix/uterine/ovarian/vulvar	38 (3%)
Colorectal	36 (3%)
Melanoma	17 (1%)
Liver	14 (1%)
Bladder	13 (0.9%)
Thyroid	13 (0.9%)
Kidney	12 (0.9%)
Other blood disorder	11 (0.8%)
Brain or central nervous system	9 (0.6%)
Pancreas	9 (0.6%)
Esophagus	8 (0.6%)
Sarcoma	8 (0.6%)
Stomach	8 (0.6%)
Oral	5 (0.4%)
Hodgkin lymphoma	3 (0.2%)
Testis	2 (0.1%)
Non-invasive tumor	2 (0.1%)
Other	72 (5%)
Missing	(11%)

*N* = 1396; ^*^ If the second digit of patients’ postal code was a zero, it was coded as rural; otherwise, it was coded as urban

### Multiple imputation for missing data

Missing data rate ranged from 0.8 to 5.0%; the question, “During the past 4 weeks, have you had any of the following problems with your work or other regular daily activities as a result of your physical health?”, accounted for the most missing values (5%). The results from Little’s MCAR test suggest that the data may be missing completely at random (X^2^ = 1.48, df = 3, *p* = 0.69). The highest proportion of missing values for the variables of interest was 5%; as a rule of thumb, the number of imputations was set at twice the maximum percentage of missing data cases (10 imputations). Binary variables were imputed using a logistic regression. Binary variables considered were education level (high school or less vs. post-secondary education); ethnicity (non-white vs. white); hospitalization in the past six months (yes/no); perceived involvement level of their healthcare provider during the pandemic (yes/no); and perceived level of cancer services received during the pandemic (yes/no).

### Patients’ physical and mental health

Table 2 describes the patients’ PCS and MCS before and during the COVID-19 pandemic. Overall, the patients’ PCS (mean ± SD) significantly decreased during the pandemic (40.8 ± 11.9) compared to before the pandemic (42.2 ± 20.8) (*p* < 0.001). There was no statistically significant change in MCS detected between the two timepoints (before the pandemic: 50.4 ± 10.1 and during the pandemic: 50.7 ± 10.4; *p* = 0.31).

**Table 2:** Patients’ physical and mental component scores before and during the COVID-19 pandemic

	Timepoint	Mean (SD)	*p*-value
Physical component score (PCS)	During the pandemic	40.77 (11.92)	< 0.0001
	Before the pandemic	42.22 (20.80)	
Mental component score (MCS)	During the pandemic	50.67 (10.39)	0.3116
	Before the pandemic	50.35 (10.13)	

*N* = 1396

In our study, individuals’ PCS was influenced by various factors, including sociodemographic variables (e.g., current age and residing in urban locations), clinical variables (e.g., time since recent cancer diagnosis, self-reported hospitalization), and telehealth experiences (e.g., number of telehealth visits). Additionally, pre-pandemic scores for both MCS and PCS played a role (Table 3). Specifically, lower PCS was associated with older age, higher number of telehealth visits, self-reported hospitalization, and a longer period since their last cancer diagnosis. Individuals with higher PCS were more likely to reside in urban areas, report higher MCS before the pandemic, have higher PCS before the pandemic, and indicate a decent perceived level of cancer services received during the pandemic.

**Table 3:** Factors affecting physical component scores during the COVID-19 pandemic

	Estimate	SE	Statistic	DF
Intercept	17.207	2.687	6.405	1321.835
Age*	−0.173	0.024	−7.184	1324.921
Female	0.844	0.497	1.697	1327.425
Urban**	1.731	0.911	1.899	1326.948
White	−0.165	0.650	−0.254	1084.951
College or more	0.745	0.508	1.467	1313.878
More than one cancer diagnosis	−0.170	0.573	−0.297	1327.223
Health authority (ref: Interior Health)				
Fraser health	−0.597	0.20	−0.829	1326.463
Vancouver Coastal health	1.176	0.790	1.488	1322.952
Vancouver Island health	0.497	0.704	0.706	1327.594
Northern health	0.722	1.304	0.554	1327.229
MCS before the pandemic*	0.061	0.023	2.622	1327.160
PCS before the pandemic*	0.659	0.023	29.199	1327.023
Perceived assessment of HCP involvement in their care	0.041	0.749	0.054	1107.014
Perceived assessment of receipt of all needed services*	3.698	1.473	2.510	983.585
Self-reported no hospitalization*	−2.807	0.56	−3.715	1274.052
Number of telehealth visits during the pandemic*	−0.124	0.036	−3.458	1327.067
Days since last diagnosis**	−0.0004	0	−2.396	1327.870
Receiving IV chemotherapy	−0.513	0.583	−0.880	1327.870

*N* = 1396; *p ≤ 0.01; **p ≤ 0.05

Similar variables also impacted patients’ MCS (Table 4). Lower MCS was associated with individuals being female and reported a higher number of telehealth visits during the pandemic. Higher MCS were reported by individuals who were white, received college or higher education, reported high MCS before the pandemic, and perceived their healthcare provider (HCP) to be involved in their care.

**Table 4:** Factors affecting mental component scores during the COVID-19 pandemic

	Estimate	SE	Statistic	DF
Intercept	15.584	2.631	5.922	1329.546
Age	0.019	0.024	0.773	1328.536
Female*	−1.277	0.489	−2.614	1330.306
Urban	1.041	0.901	1.155	1328.669
White**	1.288	0.643	2.001	1061.195
College or more*	1.402	0.501	2.801	1323.203
More than one cancer diagnosis	−0.379	0.565	−0.670	1330.444
Health authority (ref: Interior health)				
Fraser health	−0.205	0.711	−0.288	1329.910
Vancouver Coastal health	−0.923	0.780	−1.184	1326.411
Vancouver Island health	−0.026	0.696	−0.037	1330.752
Northern health	0.525	1.288	0.408	1330.817
MCS before the pandemic*	0.563	0.024	23.814	1330.067
PCS before the pandemic	0.040	0.021	1.879	1330.506
Perceived assessment of HCP involvement in their care*	2.979	0.728	4.092	1240.132
Perceived assessment of receipt of all needed services	−0.055	1.460	−0.038	1320.151
Number of telehealth visits during the pandemic*	−0.098	0.033	−2.980	1330.869
Days since last diagnosis	−0.0001	0	−0.841	1330.143

*N* = 1396; *p ≤ 0.01; **p < 0.05

## Discussion

Our study provides valuable insights into the impact of the COVID-19 pandemic on the HRQL of cancer patients in BC. Employing a unique pre-post analysis within the patient population itself, our study observed a significant reduction in PCS during the pandemic. Although this reduction was minor, as indicated in Table 2, it underscores the challenges encountered by this demographic. Influencing factors included older age, recent cancer diagnosis, self-reported hospitalization, and number of telehealth visits. Surprisingly, MCS remained stable, suggesting effective coping mechanisms. Demographic factors, such as gender and mental health status, played a role, emphasizing the need for targeted interventions for females and those with pre-existing mental health conditions.

The reduction in PCS during the pandemic compared to pre-pandemic levels aligns with public health guidance encouraging individuals to stay at home, which led to limited physical activity opportunities due to the closure of gyms and recreational centres, which in turns could result in lower HRQL [10, 11]. Alternatively, it is conceivable that these cancer patients were naturally experiencing a decline due to their cancer diagnosis or other underlying physical health issues, seemingly independent of the pandemic itself.

The observed stability in MCS among our sample of cancer patients, on the other hand, suggests that these individuals may have developed effective coping mechanisms over the course of their treatment, contributing to their resilience during the pandemic. It’s also plausible that limitations in social activities, reduced work hours, or diminished accomplishments due to cancer may have already influenced the scores for items affecting MCS. Therefore, changes resulting from the pandemic might not further impact these scores.

Our study also delved into the factors influencing individuals’ PCS and MCS during the COVID-19 pandemic, revealing a multifaceted interplay of sociodemographic, clinical, and telehealth-related variables. Common factors influencing both PCS and MCS include *number of telehealth visits, perceived assessment of HCP involvement in their care, and pre-pandemic MCS scores*. Number of telehealth visits are associated with lower scores in both PCS and MCS, underscoring potential limitations in addressing both physical and mental aspects of care. Further research and targeted interventions to address disparities may help answer important questions about the effectiveness and accessibility of telehealth services in meeting both physical and mental health needs, thus enhancing care provided through telehealth.

Perceived HCP involvement emerges as a common factor emphasizing the importance of patient-provider interactions for physical and mental well-being. Patients who perceive active involvement from their HCPs may experience a sense of support, understanding, and continuity in their care, contributing to enhanced mental health resilience [12, 13]. Furthermore, pre-pandemic MCS scores are identified as determinants for both mental and physical well-being, highlighting the interconnected nature of mental and physical health trajectories during the COVID-19 pandemic among cancer patients. Results from a larger study encompassing 19,763 adults, [14] contributes additional insights into the impact of pre-existing mental health on physical and mental well-being during the pandemic.

Lower PCS scores are associated with older age, indicating potential age-related challenges or healthcare needs. Clinical variables such as the time since recent cancer diagnosis and self-reported hospitalization significantly contribute to variations in PCS, suggesting cumulative impacts of cancer-related factors over time. Urban residence, and higher scores in both MCS and PCS before the pandemic are significant determinants of physical well-being.

Gender differences, with females experiencing lower MCS, highlight the potential gender-based impact of the pandemic and changes in healthcare delivery. Factors associated with higher MCS, including being white, having higher education, reporting high MCS before the pandemic, and perceiving active HCP involvement, signify protective elements against declines in mental health.

Longitudinal studies support our findings of no clinically meaningful change in mental health status and HRQL during the pandemic [15]. More specifically, they revealed a statistically small overall increase in symptoms, notably during the early stages (March-April, 2020) of the pandemic, followed by a significant decrease in severity from May to July, 2020 [16]. This pattern suggests an initial acute response to the unexpected event followed by psychological adaptation, as reflected in the subsequent decrease in symptom severity [16]. Given that our survey compared results from wave 2 (administered January to May 2020) and wave 3 (administered between May 1st to July 30th, 2021), the absence of immediate mental health issues following the pandemic is expected. A future study comparing wave 1 (April to June 2019) and wave 2 data may provide insight into the immediate mental health issues in our population.

## Limitations

While this study contains a large number of patients’ perspectives regarding their HRQL before and during the COVID-19 pandemic, it is important to acknowledge potential limitations. We recognize that our survey may not have captured the views of those who experience a disproportionate burden of health and social inequities, such as unstable housing, food insecurity, and mental health and substance use challenges. These individuals may face unique and amplified challenges during the pandemic, which were not fully represented in our study.

Additionally, we acknowledge that our survey was only available in English and the three most commonly spoken non-English languages in BC. This may have resulted in the exclusion of important and divergent perspectives of patients who were unable to complete the survey due to language barriers. Lastly, the ethnic distribution in our sample did not accurately reflect the diversity of ethnicities in BC, which could potentially constrain the generalizability of our findings.

## Conclusion

This study emphasizes the interconnectedness of mental and physical well-being and underscores the role of demographic factors and mental health status in understanding the comprehensive impact of the pandemic on cancer patients. The findings contribute valuable insights into the varied and prolonged mental health trajectories during the pandemic, emphasizing the need for further investigation to discern specific factors underlying stability in MCS and explore potential interventions to support the mental well-being of cancer patients in challenging circumstances.

As we navigate towards a new normal or a state of equilibrium, the challenges faced by cancer patients persist in the post-pandemic era. Considering the substantial impact of pre-pandemic mental well-being on both mental and physical well-being during the pandemic, it emphasizes the crucial need for comprehensive interventions that prioritize mental well-being among this vulnerable population before encountering another major event like the COVID-19 pandemic. Essential elements of these interventions should encompass strategies to strengthen patient-provider relationships and optimize healthcare support systems, such as telehealth services. Recognizing and addressing the nuances of these interactions can not only mitigate the impact of stressors on mental health but also contribute to a more patient-centered and supportive healthcare environment for individuals navigating the complexities of cancer treatment. Moreover, future research should aim to incorporate more inclusive survey methods to ensure a comprehensive understanding of the experiences and HRQL of all cancer patients, particularly those facing additional social determinants of health. It is also recommended to keep a record of other co-occurring conditions, such as contracting COVID-19, to control for them in the analysis, as they may have a significant impact on HRQL during COVID-19 pandemic.

## Data Availability

The datasets generated and/or analysed during the current study are not publicly available to protect the confidentiality and privacy of the study participants but are available from the corresponding author on reasonable request.

## Abbreviations

BC: British Columbia
CON: Community oncology networks
COVID-19: Coronavirus disease of 2019
HRQL: Health-related quality of life
MCAR: Missing completely at random
MCS: Mental component score
MICE: Multivariate imputation by chained equations
OCC: Outpatient cancer care
PCS: Physical component score
SAS: Statistical analysis system
SD: Standard deviations
SPSS: Statistical package for the social sciences
VR-12: Veterans RAND 12-item health survey
